# Prolonged Airway Obstruction after Posterior Occipitocervical Fusion: A Case Report and Literature Review

**DOI:** 10.4061/2011/791923

**Published:** 2011-06-30

**Authors:** Masahiro Morita, Masuhiro Nobuta, Hirotsune Naruse, Hiroaki Nakamura

**Affiliations:** ^1^Department of Orthopaedic Surgery, Izumi Municipal Hospital, 4-10-10 Fuchu Izumi City, Osaka 594-0071, Japan; ^2^Department of Neurosurgery, Izumi Municipal Hospital, 4-10-10 Fuchu Izumi City, Osaka 594-0071, Japan; ^3^Department of Orthopaedic Surgery, Osaka City University Graduate School of Medicine, 1-5-7 Asahi Abeno, Osaka 545-0051, Japan

## Abstract

The purpose of this paper was to inform the reader that prolonged upper airway obstruction after posterior cervical spine surgery is a possible complication for patients with metastatic tumor of upper cervical spine. A 49-year-old man presented severe neck pain during posture changes due to metastatic spinal tumor of C2. Occipitocervical fusion following removal of the posterior arch of C1 and laminectomy of C2 via the single posterior approach was performed 2 weeks after radiation therapy. After the surgery, life-threatening airway obstruction due to pharyngeal oedema occurred immediately after extubation that required emergency tracheostomy. The airway obstruction did not improve well during the patient's postoperative course. Once pharyngeal oedema occurs in patients with metastatic tumor of upper cervical spine who undergo posterior cervical spine surgery following radiation therapy to the neck, the pharyngeal oedema may be constant for a long period of time.

## 1. Introduction

Airway obstruction is a well-recognized complication after neck surgery, including cervical spine surgery. The airway obstruction related to neck surgery could occur due to wound haematoma, pharyngeal oedema, graft dislodgment, asthma, and other causes [1].

This condition usually develops slowly over a few hours, and its onset is unpredictable. We report a case of acute and prolonged airway obstruction in a patient who had undergone occipitocervical fusion via the single posterior approach for a metastatic spinal tumor following radiation therapy to the neck with a review of the literature. Although there are some case reports that describe life-threatening airway obstruction occurring immediately after extubation [2–5], this study is the first to describe prolonged airway obstruction occurred immediately after surgery for metastatic tumor of upper cervical spine.

## 2. Case Report

A 49-year-old man was referred to our hospital with a four-month history of severe neck pain during posture changes, as well as mild paresthesia in his left arm. Because of his severe neck pain, he could not walk or remain standing or sitting. He had undergone surgery, and chemotherapy for gastric cancer 19 months previously. Metastases to the left adrenal gland and diaphysis of the right femur were diagnosed after he received chemotherapy. Physical examination was normal except for exaggerated deep tendon reflexes in his upper and lower extremities.

**Figure 1:**
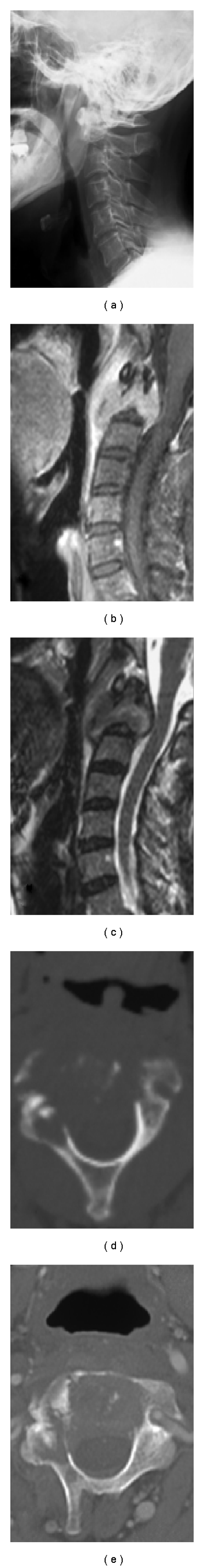
(a) Lateral radiograph showing irregular osteolysis at the anterior part of C2. Magnetic resonance (MR) imaging ((b) T1-weighted and fat suppression sagittal image with gadolinium enhancement, (c) T2-weighted sagittal image) showing a mass lesion at C2, which has marked enhancement after intravenous injection of gadolinium-DTPA. (d), (e) Axial image of computed tomography (CT) at the level of C2 showing irregular osteolysis at the anterior part of C2 and thickening of the posterior pharyngeal wall. ((d) CT for radiation therapy planning, (e) CT postradiation therapy with intravenous injection of gadolinium-DTPA).

Radiography and computed tomography (CT) of his cervical spine showed irregular osteolysis at C2, and magnetic resonance (MR) imaging revealed a mass lesion with an apparent intensity change occupying the entire anterior part of C2. The mass had marked and homogeneous enhancement after intravenous injection of gadolinium-DTPA (Figure 1). Further investigations, including technetium scintigraphy and MR imaging of the whole spine, revealed no evidence of other spinal involvement. The metastatic tumor of C2 was thought to be a candidate for his severe neck pain during posture changes, and surgical stabilization for the lesion was planned.

The patient received radiotherapy to the neck with a total dose of 30 Gy. Because his severe neck pain during posture changes had been taken worse in spite of the radiation therapy, he required surgical intervention despite his refusal at first. The preoperative anaesthetic assessment was not suggestive of any risk for airway obstruction. 2 weeks after the radiation therapy, occipitocervical fusion (C0-5) following removal of the posterior arch of C1 and laminectomy of C2 via the single posterior approach was performed. 

During the procedure for decompression and pedicle screw insertion, the upper cervical spine was fixed with a Mayfield clamp in a flexed position to obtain a wide view of the C1 posterior arch. Before fixation, the flexed position of the cervical spine was changed to obtain an adequate fusion angle using the X-ray image intensifier. The operation was performed without any significant events, though hemorrhage from the tumor located at the edge of C2 laminectomy and venous plexus surrounding that was difficult to control. The operation time was 6 hours 42 minutes, the estimated blood loss was 2900 mL, and intraoperative transfusion of red cell concentrate (8 units), fresh frozen plasma (8 units), and platelet concentrate (20 units) was performed.

Although his face and neck were edematous after surgery, he was extubated in the operating room with an adequate weaning profile and clear consciousness. However, immediately after extubation, the patient was noted to be making respiratory effort, but he appeared to be obstructed. Oxygen supply with a facemask was not effective, and reintubation using rocuronium bromide was impossible to perform due to the severe swelling of the retropharyngeal soft tissue and upper airway. Oxygen saturation started to decrease to less than 30% within a few minutes. In view of the urgency of the situation, the injection needles (18 G) were inserted below the cricoid cartilage. With oxygen supplied through the needles, oxygen saturation started to increase quickly to normal, and then emergency tracheostomy was performed. He was admitted to the intensive care unit to control his breathing by continuing sedation and continuous positive airway pressure. On postoperative day 4, he could breathe by himself thorough a tracheostomy tube without mechanical ventilation assistance.

**Figure 2:**
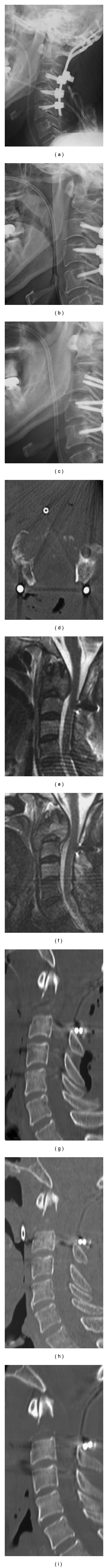
(Images of left column (a, d, g), middle column (b, e, h), and right column (c, f, i) are postoperatively 1 week, 4 weeks, and 9 weeks, resp.) Lateral radiograph (a, b, c) and axial image of CT at the level of C2 (d) showing disappearance (a, c, d) or decrease (b) of the middle pharyngeal space surrounded by the thickened pharyngeal wall. Reconstructed sagittal CT 1 week postoperatively (g) showing the disappearance of the middle pharyngeal space, that 4 weeks postoperatively (h) shows improvement and that 9 weeks postoperatively (i) shows deterioration. T2-weighted sagittal MR imaging 9 weeks postoperatively (f) showing enlargement of the mass lesion at C2 compared to that at 4 weeks postoperatively (e).

Lateral radiography and CT of his neck showed disappearance of the middle pharyngeal space along with thickening of the pharyngeal wall. Fiberscopic examination of the airway also showed reduction of the middle pharyngeal space. These findings did not improve well during the patient's postoperative course (Figure 2). A trial of weaning from the tracheostomy tube at 6 weeks postoperatively failed because of airway blockage by sputum after removal of the tracheostomy tube. Furthermore, the patient continued to require a stomach tube, because of dysphagia caused by severe pharyngeal pain on swallowing solid food or drinking water that had been observed postoperatively. However, the patient's severe neck pain during posture changes had improved postoperatively, and he could walk with a walker or move on a wheelchair until severe right neck pain, thought to be caused by C2 radiculopathy, occurred 2 months postoperatively. The patient died 4 months after the fusion surgery because of multiple organ failure caused by metastases. 

## 3. Discussion

The reported rate of postoperative airway obstruction following cervical spine surgery varies in the English literature. Sagi et al. [1] reported a rate of 4.2% (13/311) for postoperative reintubation after surgery on the anterior cervical spine. Wattenmaker et al. [6] reported a rate of 7.8% (10/128) for postoperative airway obstruction and 3.9% (5/128) for reintubation among patients with rheumatoid arthritis undergoing posterior cervical spine surgery.

Terao et al. [7] reported an increased incidence of emergency airway management among patients undergoing combined anterior-posterior cervical spine surgery compared with other cervical spine surgeries. The rates of reintubation and prophylactic delayed extubation were 30% (3/10) and 40% (4/10), respectively, after combined anterior-posterior cervical spine surgery, whereas they were 1% (2/155) and 0% (0/155), respectively, after other cervical spine surgery. On the other hand, Epstein et al. [8] reported a rate of 6.9% (4/58) for postoperative airway complications (reintubation or tracheostomy) after combined anterior-posterior cervical spine surgery. This decreased rate may be related to their protocol for extubation, which requires prophylactic intubation for at least the first postoperative night, checking residual tracheal and/or vocal cord swelling by fiberoptic bronchoscopy before extubation, and so on.

There are several risk factors that could induce airway obstruction after cervical spine surgery. Wattenmaker et al. [6] found that airway obstruction is a frequent complication after a posterior operation on the cervical spine in a patient who has rheumatoid arthritis and who is intubated without fiberoptic assistance. 

Epstein et al. [8] identified several risk factors in their comparison study between delayed extubation or tracheostomy and extubation on postoperative day one, among patients who underwent combined anterior-posterior cervical spine surgery. The risk factors they identified were long operative time (greater than 10 hours), obesity greater than 220 lbs, reoperation of anterior corpectomy and fusion, anterior corpectomy and fusion including C2, transfusion of more than 4 U of blood, anterior corpectomy and fusion of more than 4 levels, and asthma. Similar to this case control study, Kwon et al. [9] also noted several risk factors in their comparison study between delayed extubation and extubation on postoperative day one among patients who underwent combined anterior-posterior cervical spine surgery. Their data showed that individual patient factors such as age, weight, smoking, past medical history, and American Society of Anesthesiologists classification were not strongly related to the risk of delayed extubation. The statistically significant risk factors were total operative time and total volume of intraoperative intravenous fluid administration and blood replacement.

It is known that the pharyngeal airway space increases following head extension and is wider when the extension occurs at the uppermost part of the cervical spine [10, 11]. Tagawa et al. [12] reported a case presented upper airway obstruction associated with flexed cervical position after posterior occipitocervical fusion, and Miyata et al. [13] suggested that an occipito-C2 fusion angle in a flexed position was another risk factor for airway obstruction.

Although the risk factors of previous reports, such as intubation without fiberoptic assistance, long operative time, and large amount of intraoperative intravenous fluid administration and blood replacement, were present in our case, we suggested that preoperative radiation therapy and the intraoperative prone position with the cervical spine in the flexed position were the most likely factors responsible for this complication. Preoperative irradiation to the neck lesion would increase the risk of airway obstruction [14, 15], and the intraoperative prone position with the cervical spine in the flexed position could cause angioedema due to jugular venous congestion. Nömayr et al. [16] investigated the MR imaging appearance of radiation-induced changes of normal cervical tissues among patients with primary neck tumor, and they found that oedema was the main radiation-induced effect. It was constant for several months, and when the total dose was 70 Gy, the oedema could be constant for more than 12 months.

Although airway obstruction due to pharyngeal oedema after cervical spine surgery had subsided within a few days or weeks among patients reported in the previous literature [6, 7, 9], indication of surgery for these patients was spondylotic myelopathy, trauma, rheumatoid arthritis, and so on. There appears to be no English literature on the duration of the pharyngeal oedema that is responsible for airway obstruction after surgery for metastatic tumor of upper cervical spine.

It is thought to be very important to maintain a patient's airway until pharyngeal oedema subsides to reduce the potential for postextubation respiratory compromise [7–9, 17]. However, spine surgeons must remember that, even though airway obstruction could be avoided by prolonged intubation, once pharyngeal oedema occurs in patients with metastatic tumor of upper cervical spine who undergo posterior cervical spine surgery following radiation therapy to the neck, the pharyngeal oedema may be constant for a long period of time.
